# Association between comorbid asthma and prognosis of critically ill patients with severe sepsis: a cohort study

**DOI:** 10.1038/s41598-021-93907-0

**Published:** 2021-07-28

**Authors:** Jinju Huang, Jurong Zhang, Faxia Wang, Jiezhu Liang, Qinchang Chen, Zhuandi Lin

**Affiliations:** 1 Intensive Care Unit, Guangzhou Panyu Central Hospital, No. 8 Fuyu East Road, Qiaonan Street, Panyu District, Guangzhou, 511400 China; 2 Zhongshan School of Medicine, Sun Yat-Sen University, Guangzhou, 510080 China

**Keywords:** Risk factors, Asthma

## Abstract

Basic research suggests some contributing mechanisms underlying asthma might at the same time benefit patients with asthma against sepsis, while the potential protective effect of comorbid asthma on prognosis of sepsis has not been well studied in clinical research. The study aimed to assess the association between comorbid asthma and prognosis in a cohort of patients admitted to intensive care unit (ICU) with severe sepsis. Patients with severe sepsis admitted to ICUs were included from the MIMIC-III Critical Care Database, and categorized as patients without asthma, patients with stable asthma, and patients with acute exacerbation asthma. The primary study outcome was 28-day mortality since ICU admission. Difference in survival distributions among groups were evaluated by Kaplan–Meier estimator. Multivariable Cox regression was employed to examine the association between comorbid asthma and prognosis. A total of 2469 patients with severe sepsis were included, of which 2327 (94.25%) were without asthma, 125 (5.06%) with stable asthma, and 17 (0.69%) with acute exacerbation asthma. Compared with patients without asthma, patients with asthma (either stable or not) had a slightly younger age (66.73 ± 16.32 versus 64.77 ± 14.81 years), a lower proportion of male sex (56.81% versus 40.14%), and a lower median SAPS II score (46 versus 43). Patients with acute exacerbation asthma saw the highest 28-day mortality rate (35.29%), but patients with stable asthma had the lowest 28-day mortality rate (21.60%) when compared to that (34.42%) in patients without asthma. Consistent results were observed in Kaplan–Meier curves with a p-value for log-rank test of 0.016. After adjusting for potential confounding, compared to being without asthma, being with stable asthma was associated with a reduced risk of 28-day mortality (hazard ratio (HR) 0.65, 95% confidence interval (CI) 0.44–0.97, p = 0.0335), but being with acute exacerbation asthma was toward an increased risk of 28-day mortality (HR 1.82, 95% 0.80–4.10, p = 0.1513). E-value analysis suggested robustness to unmeasured confounding. These findings suggest comorbid stable asthma is associated with a better prognosis in critically ill patients with severe sepsis, while acute exacerbation asthma is associated with worse prognosis.

## Introduction

Chronic comorbid conditions are common in critically ill patients, and most of them have a negative impact on survival^1,2^. Asthma is a common and noncommunicable disease of the lungs, which was estimated to affect 235 million individuals globally in 2017^3^, although the prevalence might vary between regions^4–7^. An increase in prevalence of asthma has been observed in some countries^8–11^, but the disease itself generally has a good prognosis if its symptoms are well controlled to reduce the risk of asthma exacerbations, which is consistent with the decrease in age-standardized death rates from asthma in most countries in the past two decades^12^. However, it remains unclear about the potential impact of comorbid asthma on prognosis of critically ill patients.

Sepsis is a clinical syndrome with physiologic, biologic, and biochemical abnormalities caused by a dysregulated host response to infection, which leads to life-threatening organ dysfunction^13^. About 30% of patients admitted to general intensive care units (ICUs) and about 80% of ICU patients with infection were found to meet the criteria of sepsis^14,15^, although the results might differ when different criteria were used. The management of sepsis is still challenging, with a mortality rate ranging from 10 to 50%^16–18^.

Basic research suggests some contributing mechanisms underlying asthma are essential normal host response to pathogens and thus might at the same time benefit patients with asthma against sepsis. Parasitic infection is found to improve survival from septic peritonitis by driving type 2 helper T-cell (Th2) polarization and enhancing mast cell responses to bacteria in mice^19^. Higher circulating Th2 levels are also observed in survivors than that in patients died from Staphylococcus aureus infection^20^, while Th2 immune responses is the major contributing mechanism underlying asthma^21^. In addition to Th2 pathway, studies on non-Th2 pathways also suggest potential benefits of asthma on prognosis of infection^22,23^. As the primary sensors of invading pathogens, Toll-like receptors plays a pivotal role in airway allergic inflammation^24^. Activation of interleukin-17 (IL-17) is involved in the pathology of asthma, which might play an important role in inducing neutrophil migration to the infection focus and therefore reduce the spread of infection^25^. In addition, a reverse association between asthma and sepsis has also been reported, which indicates that polymorphisms in the myosin light chain kinase gene that confers risk of severe sepsis are associated with a lower risk of asthma^26^.

However, the potential protective effect of comorbid asthma on prognosis of sepsis has not been well studied in clinical research. A study with a large sample size reveals that diseases associated with an overactive type 2/Th2 immune response including asthma are markedly and significantly underrepresented among septic patients^27^. An observational study reports comorbid asthma is associated with lower risk of sepsis-mortality^28^, which is against findings (i.e., negative effect) from previous studies^29,30^. Considering that there is limited evidence from clinical research that specially investigate the potential impact of asthma on prognosis of critically ill patients with sepsis, and that inconsistent results have been reported, the study aimed to examine the association between comorbid asthma and prognosis of critically ill patients with severe sepsis with an improved study design.

## Methods

### Data source

The study used data from the Medical Information Mart for Intensive Care (MIMIC) III (v1.4). It is a large, freely-available database comprising de-identified health-related data associated with over forty thousand patients who stayed in critical care units of the Beth Israel Deaconess Medical Center between 2001 and 2012^31^. Access to the data was approved after completing the Collaborative Institutional Training Initiative (CITI) program “Data or Specimens Only Research”. The study was exempt from approval from the institutional review board of the Massachusetts Institute of Technology (no. 0403000206) due to the retrospective design, lack of direct patient intervention, and the security schema for which the reidentification risk was certified as meeting safe harbor standards by Privacert (Cambridge, MA). Informed consent was waived for the same reason. The study was performed in accordance with the Declaration of Helsinki.

### Study population

**Figure 1:**
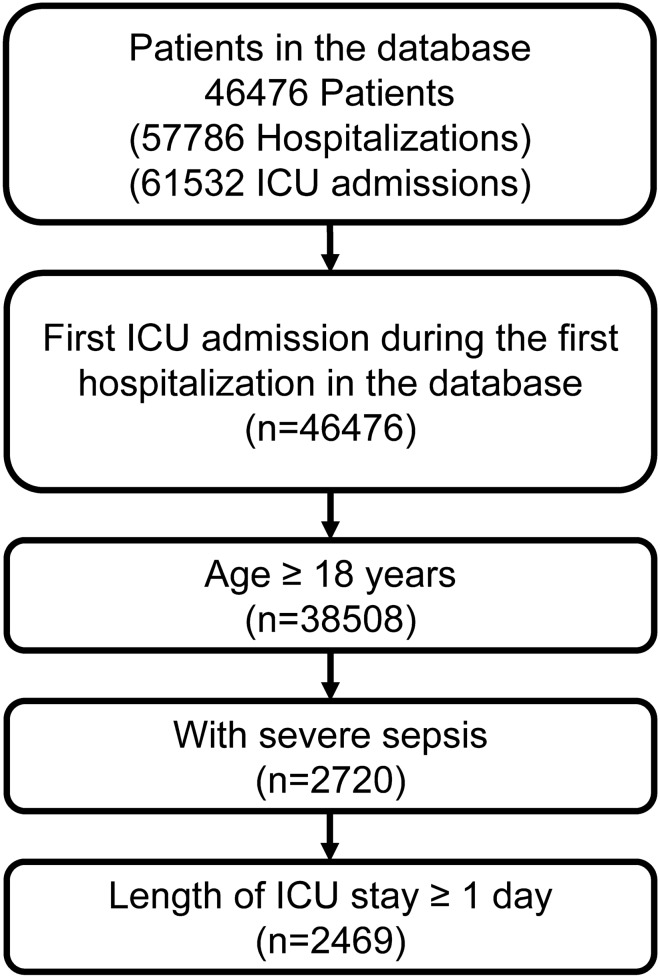
Flow chart of the patients. *ICU* intensive care unit.

The study included patients admitted to ICU with a diagnosis record of severe sepsis, which was identified by International Classification of Diseases, Ninth Revision, Clinical Modification (ICD-9-CM) code 995.92 (severe sepsis) or 785.52 (septic shock). Since a patient may have more than one hospitalization record in the database, we only included the first ICU admission during the first hospitalization in the database. Patients less than 18 years old at ICU admission and those who stayed in the first ICU hospitalization for less than 24 h were excluded. The inclusion of the patients is presented in Fig. 1.

### Exposure

The study population was categorized as three groups, namely patients without asthma, patients with stable asthma, and patients with acute exacerbation asthma. Asthma was identified based on ICD-9-CM codes. Stable asthma was identified using codes 493.00 (Extrinsic asthma, unspecified), 493.10 (Intrinsic asthma, unspecified), 493.20 (Chronic obstructive asthma, unspecified), 493.81 (Exercise induced bronchospasm), 493.82 (Cough variant asthma), and 493.90 (Asthma, unspecified type, unspecified). Acute exacerbation asthma was identified using codes 493.01 (Extrinsic asthma with status asthmaticus), 493.02 (Extrinsic asthma with (acute) exacerbation), 493.11 (Intrinsic asthma with status asthmaticus), 493.12 (Intrinsic asthma with (acute) exacerbation), 493.21 (Chronic obstructive asthma with status asthmaticus), 493.22 (Chronic obstructive asthma with (acute) exacerbation), 493.91 (Asthma, unspecified type, with status asthmaticus), and 493.92 (Asthma, unspecified type, with (acute) exacerbation).

### Outcomes

28-day mortality since ICU admission was the main study outcome, and ICU mortality was the secondary study outcome. To identify 28-day mortality, patients were followed from the date of first ICU admission to 28 days after the first ICU admission, or the date of death, whichever came first. To identify ICU mortality, patients were followed from the date of first ICU admission to date of discharge of first ICU hospitalization, or the date of death, whichever came first. Length of ICU stay (for the first ICU hospitalization) was also calculated in the study.

### Covariates

Several variables at baseline (i.e., the date of first ICU admission) were studied as covariates, including age, sex, ethnicity, marital status, type of admission (elective or urgent), Simplified Acute Physiology Score (SAPS) II, Sequential Organ Failure Assessment (SOFA) score, mechanical ventilation on first day, renal replacement therapy on first day, and various comorbidities, including congestive heart failure, cardiac arrhythmias, valvular disease, pulmonary circulation disorder, chronic obstructive pulmonary disease, peripheral vascular disorder, hypertension, paralysis, other neurological disease, uncomplicated diabetes, complicated diabetes, hypothyroidism, renal failure, liver disease, peptic ulcer, acquired immune deficiency syndrome, lymphoma, metastatic cancer, solid tumor, rheumatoid arthritis, coagulopathy, obesity, weight loss, fluid and electrolyte disorders, blood loss anemia, deficiency anemia, alcohol abuse, drug abuse, psychoses, and depression. The above variables were identified using codes from the MIMIC Code Repository^32^.

### Statistical analysis

Variables were described as mean ± standard deviation, median (25–75% percentile) or number (percentage) when appropriate. Comparisons between groups were examined by Kruskal–Wallis test or Chi-squared test (or Fisher's exact test for cells with small number)^33^. Difference in survival distributions among groups were evaluated by Kaplan–Meier estimator. Multivariable Cox regression and multivariable logistic regression were employed to examine the association of comorbid asthma with 28-day mortality and ICU mortality, respectively. We predefined two models to adjust for confounding. Model 1 was adjusting for age, sex, ethnicity, marital status, type of admission, SAPS II, mechanical ventilation on first day, and renal replacement therapy on first day. Model 2 was adjusting for Model 1 and the various comorbidities mentioned above. To evaluate the potential bias for unmeasured confounding, the E-value was calculated^34^ which quantified the required magnitude of an unmeasured confounder that could negate the observed association between comorbid asthma and prognosis. A p-value less than 0.05 is considered as statistically significant. All the analyses were performed using Empower(R) (www.empowerstats.com; X&Y solutions, Inc., Boston, MA) program.

## Results

### Demographic and clinical characteristics of the patients

A total of 2469 patients with severe sepsis were included finally. The average age of the study population was 66.62 ± 16.24 years, and 55.85% (1379/2469) were male. Among the study population, 73.31% (1810/2469) were white, and 95.95% (2369/2469) were admitted to ICU urgently; 2327 (94.25%) were without asthma, 125 (5.06%) with stable asthma, and 17 (0.69%) with acute exacerbation asthma. Compared with patients without asthma, patients with asthma (either stable or not) had a slightly younger age (66.73 ± 16.32 versus 64.77 ± 14.81 years), a lower proportion of male sex (56.81% versus 40.14%), and a lower median SAPS II score (46 versus 43). Overall, the three groups showed similar baseline characteristics, where statistically significant difference was observed only in sex, ethnicity, chronic obstructive pulmonary disease, obesity, and depression. Detailed results are presented in Table 1.

**Table 1:** Demographic and clinical characteristics of the patients.

Variables	Total (n = 2469)	Without asthma (n = 2327)	Stable asthma (n = 125)	Acute exacerbation asthma (n = 17)	p-value
Age (years)	66.62 ± 16.24	66.73 ± 16.32	64.77 ± 14.61	64.83 ± 16.70	0.380
Male	1379 (55.85%)	1322 (56.81%)	53 (42.40%)	4 (23.53%)	< 0.001
**Ethnicity**					0.016
White	1810 (73.31%)	1711 (73.53%)	90 (72.00%)	9 (52.94%)	
Black	207 (8.38%)	190 (8.17%)	14 (11.20%)	3 (17.65%)	
Hispanic	63 (2.55%)	60 (2.58%)	3 (2.40%)	0 (0.00%)	
Asian	75 (3.04%)	71 (3.05%)	1 (0.80%)	3 (17.65%)	
Other	59 (2.39%)	57 (2.45%)	1 (0.80%)	1 (5.88%)	
Unknown	255 (10.33%)	238 (10.23%)	16 (12.80%)	1 (5.88%)	
**Marital status**					0.894
Married/life partner	1104 (44.71%)	1046 (44.95%)	52 (41.60%)	6 (35.29%)	
Single/divorced/separated	818 (33.13%)	765 (32.87%)	45 (36.00%)	8 (47.06%)	
Widowed	399 (16.16%)	376 (16.16%)	21 (16.80%)	2 (11.76%)	
Unknown	148 (5.99%)	140 (6.02%)	7 (5.60%)	1 (5.88%)	
**Type of admission**					0.471
Elective	100 (4.05%)	93 (4.00%)	7 (5.60%)	0 (0.00%)	
Urgent	2369 (95.95%)	2234 (96.00%)	118 (94.40%)	17 (100.00%)	
SAPS II	46 (36–57)	46 (36–57)	43 (34–56)	43 (28–49)	0.050
SOFA	7 (5–10)	7 (5–10)	7 (5–9)	6 (4–8)	0.284
Mechanical ventilation on first day	1384 (56.06%)	1302 (55.95%)	75 (60.00%)	7 (41.18%)	0.312
Renal replacement therapy on first day	172 (6.97%)	162 (6.96%)	9 (7.20%)	1 (5.88%)	0.979
**Comorbidities**					
Congestive heart failure	866 (35.07%)	822 (35.32%)	37 (29.60%)	7 (41.18%)	0.370
Cardiac arrhythmias	854 (34.59%)	810 (34.81%)	41 (32.80%)	3 (17.65%)	0.304
Valvular disease	268 (10.85%)	251 (10.79%)	15 (12.00%)	2 (11.76%)	0.907
Pulmonary circulation disorder	178 (7.21%)	167 (7.18%)	9 (7.20%)	2 (11.76%)	0.767
Chronic obstructive pulmonary disease	297 (12.03%)	296 (12.72%)	1 (0.80%)	0 (0.00%)	< 0.001
Peripheral vascular disorder	244 (9.88%)	230 (9.88%)	13 (10.40%)	1 (5.88%)	0.842
Hypertension	1294 (52.41%)	1223 (52.56%)	63 (50.40%)	8 (47.06%)	0.811
Paralysis	95 (3.85%)	90 (3.87%)	4 (3.20%)	1 (5.88%)	0.846
Other neurological disease	309 (12.52%)	287 (12.33%)	21 (16.80%)	1 (5.88%)	0.241
Uncomplicated diabetes	605 (24.50%)	568 (24.41%)	32 (25.60%)	5 (29.41%)	0.855
Complicated diabetes	180 (7.29%)	172 (7.39%)	7 (5.60%)	1 (5.88%)	0.736
Hypothyroidism	270 (10.94%)	246 (10.57%)	21 (16.80%)	3 (17.65%)	0.063
Renal failure	467 (18.91%)	441 (18.95%)	25 (20.00%)	1 (5.88%)	0.371
Liver disease	356 (14.42%)	335 (14.40%)	20 (16.00%)	1 (5.88%)	0.533
Peptic ulcer	3 (0.12%)	3 (0.13%)	0 (0.00%)	0 (0.00%)	1.000
Acquired immune deficiency syndrome	46 (1.86%)	45 (1.93%)	1 (0.80%)	0 (0.00%)	0.560
Lymphoma	79 (3.20%)	78 (3.35%)	0 (0.00%)	1 (5.88%)	0.095
Metastatic cancer	188 (7.61%)	178 (7.65%)	8 (6.40%)	2 (11.76%)	0.711
Solid tumor	131 (5.31%)	124 (5.33%)	7 (5.60%)	0 (0.00%)	0.614
Rheumatoid arthritis	88 (3.56%)	82 (3.52%)	5 (4.00%)	1 (5.88%)	0.841
Coagulopathy	786 (31.83%)	744 (31.97%)	37 (29.60%)	5 (29.41%)	0.838
Obesity	171 (6.93%)	150 (6.45%)	19 (15.20%)	2 (11.76%)	< 0.001
Weight loss	226 (9.15%)	214 (9.20%)	11 (8.80%)	1 (5.88%)	0.886
Fluid and electrolyte disorders	1390 (56.30%)	1315 (56.51%)	66 (52.80%)	9 (52.94%)	0.690
Blood loss anemia	65 (2.63%)	65 (2.79%)	0 (0.00%)	0 (0.00%)	0.130
Deficiency anemia	700 (28.35%)	654 (28.10%)	39 (31.20%)	7 (41.18%)	0.378
Alcohol abuse	206 (8.34%)	194 (8.34%)	11 (8.80%)	1 (5.88%)	0.919
Drug abuse	82 (3.32%)	75 (3.22%)	6 (4.80%)	1 (5.88%)	0.530
Psychoses	94 (3.81%)	86 (3.70%)	8 (6.40%)	0 (0.00%)	0.218
Depression	213 (8.63%)	186 (7.99%)	23 (18.40%)	4 (23.53%)	< 0.001

*SAPS II* simplified acute physiology score II, *SOFA* sequential organ failure assessment.

### Clinical outcomes of the patients

**Figure 2:**
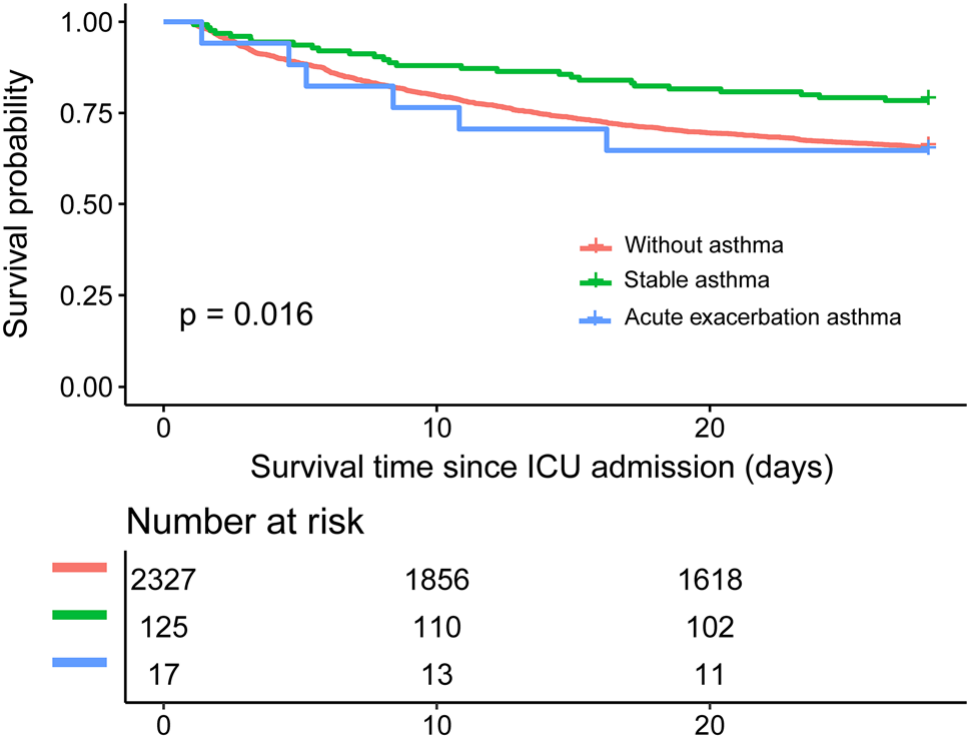
Kaplan–Meier curves of the patients. *ICU* intensive care unit.

As presented in Table 2, patients with acute exacerbation asthma saw the highest 28-day mortality rate (35.29%, 6/17), but patients with stable asthma had the lowest 28-day mortality rate (21.60%, 27/125) when compared to that (34.42%, 801/2327) in patients without asthma. Consistent results were observed in Kaplan–Meier curves with a p-value for log-rank test of 0.016 (Fig. 2). Similar to 28-day mortality, the highest ICU mortality was seen in patients with acute exacerbation asthma (41.18%, 7/17), and the lowest was seen in patients with stable asthma (16.00%, 20/125). There was no statistically significant difference in the length of ICU stay between groups (p = 0.714).

**Table 2:** Clinical outcomes of the patients.

Variables	Total (n = 2469)	Without asthma (n = 2327)	Stable asthma (n = 125)	Acute exacerbation asthma (n = 17)	p-value
28-day mortality	834 (33.78%)	801 (34.42%)	27 (21.60%)	6 (35.29%)	0.013
ICU mortality	605 (24.50%)	578 (24.84%)	20 (16.00%)	7 (41.18%)	0.023
Length of ICU stay (days)	4.98 (2.55–11.54)	4.95 (2.55–11.46)	5.67 (2.16–12.39)	5.49 (3.62–11.84)	0.714

*ICU* intensive care unit.

### Association between comorbid asthma and prognosis in the patients

After adjusting for potential confounding, compared to being without asthma, being with stable asthma was associated with a reduced risk of 28-day mortality (hazard ratio (HR) 0.65, 95% confidence interval (CI) 0.44–0.97, p = 0.0335, adjusting for Model 2), but being with acute exacerbation asthma was toward an increased risk of 28-day mortality (HR 1.82, 95% 0.80–4.10, p = 0.1513, adjusting for Model 2). Results for ICU mortality were similar, which showed being with stable asthma was associated with a reduced risk of ICU mortality (odds ratio (OR) 0.57, 95% CI 0.33–0.98, p = 0.0406), but being with acute exacerbation asthma was statistically significantly associated with an increased risk of ICU mortality (OR 4.01, 95% CI 1.35–11.97, p = 0.0127). Detailed results are presented in Tables 3 and 4. The primary findings of the associations between comorbid stable asthma and the studied clinical outcomes were robust, unless there was an unmeasured confounding factor associated with 28-day mortality with an adjusted HR of 2.03 or an unmeasured confounding factor associated with ICU mortality with an adjusted OR of 1.98.

**Table 3:** Association between comorbid asthma and 28-day mortality in the patients.

	Hazard ratio (95% CI)	p-value
**Crude**		
Without asthma	1 (Reference)	
Stable asthma	0.58 (0.39–0.84)	0.0047
Acute exacerbation asthma	1.06 (0.48–2.37)	0.8846
**Model 1**		
Without asthma	1 (Reference)	
Stable asthma	0.63 (0.43–0.92)	0.0175
Acute exacerbation asthma	1.39 (0.62–3.11)	0.4237
**Model 2**		
Without asthma	1 (Reference)	
Stable asthma	0.65 (0.44–0.97)	0.0335
Acute exacerbation asthma	1.82 (0.80–4.10)	0.1513

Model 1 was adjusting for: age, sex, ethnicity, marital status, type of admission, SAPS II, mechanical ventilation on first day, and renal replacement therapy on first day. Model 2 was adjusting for: Model 1 and various comorbidities, including congestive heart failure, cardiac arrhythmias, valvular disease, pulmonary circulation disorder, chronic obstructive pulmonary disease, peripheral vascular disorder, hypertension, paralysis, other neurological disease, uncomplicated diabetes, complicated diabetes, hypothyroidism, renal failure, liver disease, peptic ulcer, acquired immune deficiency syndrome, lymphoma, metastatic cancer, solid tumor, rheumatoid arthritis, coagulopathy, obesity, weight loss, fluid and electrolyte disorders, blood loss anemia, deficiency anemia, alcohol abuse, drug abuse, psychoses, and depression.

*CI* confidence interval.

**Table 4:** Association between comorbid asthma and ICU mortality in the patients.

	Hazard ratio (95% CI)	p-value
**Crude**		
Without asthma	1 (Reference)	
Stable asthma	0.58 (0.35–0.94)	0.0267
Acute exacerbation asthma	2.12 (0.80–5.59)	0.1296
**Model 1**		
Without asthma	1 (Reference)	
Stable asthma	0.58 (0.35–0.98)	0.0399
Acute exacerbation asthma	3.56 (1.23–10.29)	0.0192
**Model 2**		
Without asthma	1 (Reference)	
Stable asthma	0.57 (0.33–0.98)	0.0406
Acute exacerbation asthma	4.01 (1.35–11.97)	0.0127

Model 1 was adjusting for: age, sex, ethnicity, marital status, type of admission, SAPS II, mechanical ventilation on first day, and renal replacement therapy on first day. Model 2 was adjusting for: Model 1 and various comorbidities, including congestive heart failure, cardiac arrhythmias, valvular disease, pulmonary circulation disorder, chronic obstructive pulmonary disease, peripheral vascular disorder, hypertension, paralysis, other neurological disease, uncomplicated diabetes, complicated diabetes, hypothyroidism, renal failure, liver disease, peptic ulcer, acquired immune deficiency syndrome, lymphoma, metastatic cancer, solid tumor, rheumatoid arthritis, coagulopathy, obesity, weight loss, fluid and electrolyte disorders, blood loss anemia, deficiency anemia, alcohol abuse, drug abuse, psychoses, and depression.

*ICU* intensive care unit, *CI* confidence interval.

## Discussion

In the study, we assessed the associations between comorbid asthma and prognosis of ICU patients with severe sepsis, indicating that comorbid stable asthma is associated with better prognosis in ICU patients with severe sepsis, while comorbid acute exacerbation asthma is associated with worse prognosis. Our study for the first time examined and distinguished the difference in the roles between stable asthma and acute exacerbation asthma, which increases knowledge in this field. It suggests that there could be potential benefit of comorbid asthma on prognosis of sepsis, but the potential protective effect might only exist in patients with stable asthma.

Based on results in our study, for ICU patients with severe sepsis, when compared to those without asthma, the risk of 28-day mortality decreases by more than 30% in those with stable asthma, and the risk of ICU mortality decreases by more than 40%. Considering the prognosis of sepsis especially severe sepsis is yet to improve^17^, this finding is, to some extent, inspiring. It suggests that as what we speculated from basic researches, the mechanisms underlying asthma at the same time provides a protective effect on patients suffering from severe infection^24,25,35^. This gives future researches a direction, and it is promising to improve prognosis by avoiding sepsis-related outcomes in people without asthma when the underlying mechanisms are better understood. Our findings are consistent with a few currently available clinical researches, although some differences between these studies should be noticed. The study from Krishack et al^27^ found the prevalence of asthma in the septic patients was lower than that in the non-septic patients (14% versus 19%). In our study, the prevalence of asthma in the patients with severe sepsis was 5.8%, which was also lower than that in the general ICU patients (6.7%)^36^, but it should be noted that the patient population investigated in the study by Krishack et al.^27^ was not restricted to ICU patients only. The study from Zein et al.^28^ included a much larger sample size compared with ours by using five independent datasets, but only age, sex, ethnicity, income, and comorbidities were considered as confounding factors when assessing the association. Our study specially included patients with severe sepsis only, which could be a reason for a much higher average age (66 versus 55 years) of the study population when compared to Zein et al.’s study^28^. To identify sepsis by ICD codes has been proved to be a challenge, so we only included patients with severe sepsis since the two ICD codes we used for identification of severe sepsis have been reported to have a positive predictive value of 100% and a specificity of 100%^37^. Our study also took severity of asthma into consideration, and results of our study indicate that unlike stable asthma, comorbid acute exacerbation asthma significantly increases the risk of ICU mortality by about 3 folds. This finding is important, because it might partly explain why controversial conclusions were drawn from different studies that investigated asthma and clinical outcomes^28,30^, although relevant evidence is still very limited. In addition, it suggests that the potential mechanisms of the protective effect could also be related to the use of medication that led to a well control of symptoms of asthma. Apart from the confounding factors considered in Zein et al.’s study^28^, the illness severity score SAPS II was also adjusted in our study, which would greatly increase the strength of our study, since it has been reported to be associated with prognosis of sepsis patients^38^ and therefore relieve the concern about potential confounding bias.

Although our study cannot provide evidence on the mechanisms underlying the findings, it is interesting to mention that the potential protective effect of comorbid asthma could also exist in other disease. A propensity-score matched cohort study found asthma was associated with lower mortality in patient with myocardial infarction^39^. The mechanism was suspected to be that Th2 responses (including IL-4 and IL-5) and non-Th2 responses (IL-17), which were involved in the pathogenesis of asthma, might be protective against atherosclerosis and myocardial infarction. Similar findings were also reported in patients with Coronavirus Disease 2019 (COVID-19). Li et al.^40^ analyzed 548 hospitalized COVID-19 patients and reported a prevalence of asthma of 0.9%, which was markedly lower than that (6.4%) in the adult population in the same region. Avdeev et al.^41^ reported a prevalence of asthma of 1.8% among 1307 ICU patients with COVID-19. Similar findings were also found in the study from Zhang et al.^42^. Although interpretation of these results should be cautious, because the study designs of these two studies were not appropriate to assess the potential impact of comorbid asthma on prognosis, they did provide some clues to research in this field.

Our study had some limitations. First, only severe sepsis patients were included, it remains unknown whether the findings can be directly applied to other conditions, such as less severe sepsis or the general ICU patients with infection. This seems possible, since a large observational study found that comorbid type 2 immune diseases (including asthma) was associated with lower risk of hospital mortality in an unselected cohort of patients Hospitalized for Acute Infection^43^. Second, although various confounding factors were considered in the study, unmeasured confounding cannot be ruled out. Due to data limitation, confounding such as smoking history and medication use were not adjusted. However, based on results of the E-value analysis, an unmeasured confounder was unlikely to explain the entirety of the observed protective effect of comorbid stable asthma. Third, measurement bias is another concern. In the study, exposure and various covariates were mainly identified by ICD codes, which was not further validated in the study due to the nature of a retrospective study design. We think this could not be a serious issue, since a female predominance was observed in the asthma patients, and there was a significantly higher proportion of comorbid obesity in the asthma patients when compared to those without asthma, which was consistent with investigations from other studies^44,45^ and suggest the quality of data used in our study was somewhat reliable. Last, the limitation on the sample size (especially for patients with acute exacerbation asthma) made further subgroup analysis unavailable in the study, but a further investigation on the association in patients with sepsis caused by different pathogens will increase the knowledge in this field.

## Conclusion

Comorbid stable asthma is associated with a better prognosis in critically ill patients with severe sepsis, while acute exacerbation asthma is associated with worse prognosis. Further researches on understanding the underlying mechanisms are warranted and may discover new intervention to improve the management of sepsis.
